# Yield reduction historically associated with the *Aegilops ventricosa* 7D^V^ introgression is genetically and physically distinct from the eyespot resistance gene *Pch1*

**DOI:** 10.1007/s00122-019-03502-1

**Published:** 2019-12-13

**Authors:** Marianna Pasquariello, Simon Berry, Christopher Burt, Cristobal Uauy, Paul Nicholson

**Affiliations:** 1 John Innes Centre, Norwich Research Park, Colney, Norwich, NR4 7UH UK; 2 Limagrain UK Ltd, Rothwell, Market Rasen, Lincolnshire, LN7 6DT UK; 3 RAGT Seeds, Grange Road, Ickleton, Essex CB10 1TA UK

## Abstract

**Key message:**

Yield penalty and increased grain protein content traits associated with *Aegilops ventricosa* 7D introgression have been mapped for the first time, and they are physically distinct from the eyespot resistance locus *Pch1.*

**Abstract:**

Wheat wild relatives represent an important source of genetic variation, but introgression of agronomically relevant genes, such as for disease resistance, may lead to the simultaneous introduction of genetically linked deleterious traits. *Pch1* is a dominant gene, conferring resistance to eyespot and was introgressed to wheat from *Aegilops ventricosa* as part of a large segment of the 7D^V^ chromosome. This introgression has been associated with a significant yield reduction and a concomitant increase in grain protein content. In this study, we evaluated both traits and their relationship to the location of the *Pch1* gene. We found that both QTLs were clearly distinct from the *Pch1* gene, being located on a different linkage group to *Pch1*. In addition, we found that the QTL for increased grain protein content was strong and consistent across field trials, whereas the yield penalty QTL was unstable and environmentally dependent. The yield and grain protein content QTLs were genetically linked and located in the same linkage group. This finding is due in part to the small size of the population, and to the restricted recombination between wheat 7D and *Ae. ventricosa* 7D^v^ chromosomes. Although recombination in this interval is rare, it does occur. A recombinant line containing *Pch1* and *7D_KASP6*, the marker associated with increase in grain protein content, but not *Xwmc221*, the marker associated with the yield penalty effect, was identified.

**Electronic supplementary material:**

The online version of this article (10.1007/s00122-019-03502-1) contains supplementary material, which is available to authorized users.

## Introduction

One of the most effective, economic, environmentally friendly and sustainable strategies to control plant diseases is the development and use of genetically resistant cultivars (Mundt 2014). The main limitation of this process is the need to continuously identify novel sources of resistance and introduce them into elite cultivars. In wheat, wild relatives represent an important source of genetic variation for disease resistance. Over 52 wheat-related species from 13 genera have been exploited as donors thanks to the plasticity of the recipient’s genome (Wulff and Moscou 2014). Although several barriers restricting interspecies hybridization and introgression have been overcome, the simultaneous introduction of genetically linked deleterious traits (i.e. linkage drag) is still a major problem hindering the effective deployment of introgressed resistance genes for crop improvement. The leaf rust resistance gene *Lr19*, transferred to wheat from *Thinopyrum ponticum*, confers a yellow colour to flour (Knott 1980) and is an example of the adverse effect on quality traits caused by resistance breeding. Moreover, dough stickiness is present in wheat lines containing the 1BL–1RS wheat–rye (*Secale cereale*) chromosome translocation originally introduced to enhance rust resistance through the action of *Yr9, Lr26* and *Sr31* (Dhaliwal et al. 1987; Hsam et al. 2000). Another introgressed gene which has been reported to have a pleiotropic negative effect is the eyespot resistance gene *Pch1* (Kwiatek et al. 2016).

Eyespot is a component of the stem-base disease complex of wheat that poses a frequent threat to winter wheat crops in Europe and the Pacific Northwest region of the USA where it can cause severe lodging and reductions in yield (Strausbaugh and Murray 1989). In Europe, the situation is particularly serious in current farming systems with a high proportion of cereal crops and a reduced crop rotation (De Boer et al. 1993). Disease is caused by two closely related fungal species *Oculimacula yallundae* and *O. acuformis* which infect wheat (*Triticum aestivum* L., *Triticum durum* L.), barley (*Hordeum vulgare* L.), oats (*Avena sativa* L.) and rye (*Secale cereale* L.). In cases of severe infection, the disease causes lodging and premature ripening of grain, leading to yield losses of up to 40% (Meyer et al. 2011).

Only three sources of resistance are known to be effective against eyespot. The most potent of these is the dominant gene *Pch1* which derives from chromosome 7D^V^ of the wheat relative *Aegilops ventricosa* and was introduced into hexaploid wheat by introgression of a large segment of 7D^V^ into chromosome 7D of wheat to produce the line ‘VPM1’ (Maia 1967). Although *Pch1* is highly effective against both eyespot species, its presence in commercial wheat varieties in Europe has been limited because of linkage drag associated with the *Ae. ventricosa* segment. A significant reduction in yield and thousand-kernel weight has been observed in *Pch1* containing lines in the absence of the disease. For example, Koen et al. (2002) found that the presence of the *Pch1* introgression segment had a detrimental effect on thousand-kernel weight in two near isogenic lines (8.5–9.25% lower than the SST66 parental line) and on yield in one line (40% less than the Palmiet parental line), but no detrimental effects on quality were detected. Kwiatek et al. (2016) also demonstrated that the presence of *Pch1* containing *Ae. ventricosa* segment caused statistically significant yield losses, both as a single eyespot resistance source or in a combination with a second eyespot resistance (*Q.Pch.jic*-*5A*), corresponding, respectively, to 10.38% and 10.51% in comparison with non-resistant control wheat varieties. Previously, Worland et al. (1990) observed a yield reduction of approximately 8% associated with the *Ae. ventricosa* introgression in recombinant lines developed from crossing VPM1 to Hobbit Sib and mapped this effect to chromosome 7D between the *Pch1/Ep*-*D1* and *LrVPM* loci.

Varieties carrying *Pch1* have been widely used in environments such as the Pacific Northwest of the USA where eyespot is severe, and the presence of the *Ae. ventricosa Pch1* containing segment is not the primary limitation on yield (Wei et al. 2011). However, in northern Europe, the perception that the presence of the *Pch1 Ae. ventricosa* segment has an adverse effect on yield has limited uptake of this resistance (Kwiatek et al. 2016). For example, it is only recently that *Pch1* carrying varieties have been developed with sufficiently high yield potential to succeed in being placed on the Agriculture and Horticulture Development Board’s Recommended List for cereals in the UK. Although recombination does occur between the *Ae. ventricosa* 7D^V^ and the wheat 7D chromosome segments, it does so at a much lower frequency than normal (Worland et al. 1988). Burt and Nicholson (2011), however, has shown that many of these varieties carry a large portion of the original *Ae. ventricosa* segment. This suggests that the negative yield effect of the 7D^V^ segment may be compensated by other factors rather than being removed in these high-yielding varieties.

The nutritional quality of wheat grains significantly impacts human health and well-being. Grain protein content is an important trait for both nutritional value and end-use quality of wheat (Veraverbeke and Delcour 2002). Protein level in modern wheat cultivars is naturally low, and improvements in the nutritional quality of wheat, such as increased protein and grain micronutrient levels are important traits to be considered in breeding programs. Producing wheat varieties high in both grain protein content (GPC) and grain yield is a major challenge in wheat breeding programs (Zhao et al. 2009), but can be achieved (Vishwakarmaa et al. 2014). The simultaneous improvement of GPC and grain yield has been hampered by the strong negative correlation between these two traits (Simmonds 1995). Some varieties, however, have a higher GPC than predicted from yield alone, and it has been shown that deviation from this negative relationship (termed grain protein deviation or GPD) has a genetic basis (Bogard et al. 2010). There is evidence that the *Ae. ventricosa Pch1* 7D^V^ segment confers a GPD of 2–3% higher grain protein content than expected on the basis of yield as well as 100–150 greater dough strength units resulting in enhanced bread making potential (Bogard et al. 2010; Groos et al. 2004).

To realize the full potential of the *Ae ventricosa* 7D^V^ introgression for eyespot resistance, it is important to determine the relationship between *Pch1*, yield and GPC. The main objective of this work was to locate the positions of the yield penalty and GPC loci on the 7D^V^ segment and determine their relation to the *Pch1* locus. This information will establish whether it is possible to separate the desirable eyespot resistance and grain protein content traits from the deleterious yield effect.

## Materials and methods

### Plant material

A BC_5_ recombinant population (RVPM7D) of 90 lines, originally produced by Worland et al. (1988), was used in the current study to define the relationship between yield-, GPC- and *Pch1*-mediated eyespot resistance. This population was developed by crossing the eyespot susceptible line Hobbit ‘sib’ (HS) and the *Pch1* containing substitution line Hobbit ‘sib’-VPM7D (HS/VPM7D). HS/VPM7D is an intergenotypic single chromosome substitution line where chromosome 7D of Hobbit ‘sib’ was replaced by chromosome 7D^v^ of *Ae. ventricosa* Tausch (2*n* = 4*x* = 28, genomes D^v^D^v^M^v^M^v^) (Doussinault et al. 1983; Maia 1967). This population was previously used by Chapman et al. (2008) to map *Pch1* to the distal end of chromosome 7D^V^.

### Field trials

A subset of 34 lines of the HSxHS/VPM7D population representing all the recombination haplotypes identified in the population was used for yield and GPC assessment across eight field trials conducted across four consecutive years (one trial in 2014, three in 2015, three in 2016 and one in 2017). These trials were carried out in the following locations of the United Kingdom:

- John Innes Centre—Church Farm, Bawburgh, Norfolk (52° 37′ 46.4″ N 1° 10′ 29.4″ E) in 2014, coded as 2014_JIC;
- RAGT Seeds Ltd, at Stapleford (52° 14′ 27.5″ N 0.16° 28′ 3″ E) and Great Shelford (52° 15′ 84.9″ N 0.14° 47′ 7.0E), Cambridgeshire, in 2015 (coded as 2015_RAGT_SF and 2015_RAGT_WH, respectively); at Elmdon, Essex (52° 07′ 09.3″ N 0.1° 05′ 98.4″ E and coded as 2016_RAGT_BT) and Ickleton, Cambridgeshire (52° 08′ 63.8″ N 0.13° 54′ 5.3″ E and coded as 2016_RAGT_SD);
- Limagrain UK Ltd, at Burnt Fen (52° 12′ 16.9″ N 0° 53′ 04.2″ E), Littleport, Ely CB7 4SU in 2015 (codes as 2015_Limagrain) and at Lower Barn (52° 11′ 56.3″ N 0° 51′ 11.1″ E), Gedding, Bury Saint Edmunds IP30 0QD in 2016 (coded as 2016_Limagrain);
- Morley St. Botolph Wymondham, Norfolk in 2017, coded as 2017_JIC.

For 2014_JIC and 2017_JIC, all entries were planted as three replications in randomized complete block designs. The plots were 4 × 1.5 m corresponding to 6 m^2^.

2015_Limagrain and 2016_Limagrain trials were randomized using an Alpha design with six replications and six sub-blocks of seven varieties/replicate. Plot size was 5.4 m^2^ with plot length of 6 m and plot width of 1.55 m.

The RAGT trials were conducted in a randomized complete block design with 2 blocks, each block containing 1 replicate. A plot size of 7.2 m^2^ (6 m × 1.2 m) was used.

All trials were run using standard agronomic packages of fertilisers, pesticides and growth regulators.

### Markers

Aiming at high-quality genomic DNA, DNA extraction of the parental lines was performed using the CTAB method (Nicholson et al. 1996). Instead a, 96-well extraction protocol adapted from Pallotta et al. (2003) was used for the DNA extraction of the populations lines.

Both HS and HS/VPM7D were genotyped by Axiom^®^ wheat HD Genotyping Array (Winfield et al. 2016). After analysing all the SNPs mapping to the 7D chromosome, a set of SNPs polymorphic between the two parental lines located across the full chromosome were selected. KASP primers were designed on these SNPs using PolyMarker (http://www.polymarker.info/) (Ramirez-Gonzalez et al. 2015). Thermodynamic properties of designed primers were verified after adding the standard FAM or HEX compatible tails (LGC ltd). 7D-specific KASP markers were initially tested against HS and HS/VPM7D, and those that were polymorphic were then assayed across the RVPM7D population.

Nine SSRs, namely *Xbarc97*, *Xgdm67*, *Xgdm86*, *Xgdm150*, *Xgwm37*, *Xgwm428*, *Xwmc14*, *Xwmc221* and *Xwmc273* and two biochemical markers, *RC3* and *Amy*-*D2*, were also included in the analysis and carried over from Chapman et al. (2008).

For the KASP assay, 2 µl (5 ng/µl) of the extracted DNA was added to 0.056 µl of primer mix (12 µl each of specific primer, 30 µl of the common primer and 46 µl deionized water) and 2 µl of KASP master mix (LGC). The PCR amplification included an initial denaturation step of 94 °C for 15 min followed by 10 cycles of touchdown PCR (annealing 62 °C to 56.6 °C, decreasing 0.6 °C per cycle), then 25 cycles of 94 °C for 10 s and 60 °C for 1 min. After amplification, plates were read into the Tecan Safire plate reader and genotyped using the Klustercaller™ software (version 2.22.0.5, LGC).

PCR reactions were prepared in a 6.25 µl final volume containing 2.5 µl DNA (10 ng/µl), 3.125 µl Taq Mastermix (Qiagen) and 0.625 µl of the relevant primer pair (2 µM). A common PCR programme was used throughout consisting of a denaturing step of 95 °C for 5 min; followed by 35 cycles of 95 °C for 30 s, 58 °C for 30 s and 72 °C for 1 min, with a final elongation step of 72 °C for 7 min. Where required, PCR products were then purified using QIAquick Gel Extraction Kit (Qiagen), sequenced using BigDye^®^ Terminator v3.1 Cycle Sequencing Kit (following the manufacturer’s instructions) and aligned in VectorNTI^®^ (ThermoScientific).

### Map construction and QTL analysis

The genetic map of chromosome 7D was generated in JoinMap© (version 3.0) (Stam 1993) using default parameters. Mapping data were combined with phenotypic data from the field for QTL analysis. Predicted mean scores were calculated for each line using a general linear model (GLM) in Genstat v.19 (Copyright 2009 Lawes Agricultural Trust, Rothamsted Experimental Station, UK). The QTL analysis was carried out using the predicted mean score data from each phenotype trial individually as well as using a data set in which the data from all trials were combined.

The identification of QTLs was done using Single Trait Linkage Analysis of Genstat v.19 (Copyright 2009 Lawes Agricultural Trust, Rothamsted Experimental Station, UK) in three different steps: (1) initial genome-wide scan by simple interval mapping (SIM) to obtain candidate QTL positions; (2) one or more rounds of composite interval mapping (CIM), in the presence of cofactors, which are potential QTL positions detected at the previous step; (3) fit the final QTL model. Default threshold based on the estimation of the effective number of tests (Li and Ji 2005) was selected for the QTL analysis.

### Phenotypic analysis

#### Thousand grain weight

TGW, grain length and grain width were performed using the Marvin grain analyser (GTA Sensorik GmbH, Neubrandenburg, Germany) using grain from the field grown BC_5_ plants.

#### Yield

JIC and RAGT field plots were harvested with Zurn 150 plot combine harvesters, which have on board weighing systems. For the JIC trials, the yield figure is the total from 6 m^2^. After machine threshing, the grain was weighed and the yields were corrected to a moisture content of 14%. The plot weights for RAGT trials were adjusted to 14% moisture content and calculated as tonnes/hectare (t/ha).

2015_Limagrain trial was harvested with a Wintersteiger Delta combine harvester, which has on board weighing system. 2016_Limagrain, instead, was harvested using a Wintersteiger Classic combine harvester. So, the whole plot was bagged on the side of the combine and then the grain weighed in the barn.

#### Grain protein content

GPC was assessed using near-infrared (NIR) spectroscopy with the method previously published by Chia et al. (2017). A FOSS 6500 wavelength scanning near-infrared microscope incorporating ISIscan™ Routine Analysis Software was used to measure protein content, moisture content and grain hardness for each sample according to the manufacturer’s instructions. Each sample of ~ 5 g of grain was run in duplicate using a ring cup. The sample spectra were compared with calibration set spectra taken from samples with known protein content, moisture and hardness compositions.

### Statistical analysis

Analysis of variance was performed on yield, grain protein content and TGW scores to assess the variation attributable to line, blocks and interactions between line and blocks, using a general linear model (GLM) in Genstat v.19 (Copyright 2009 Lawes Agricultural Trust, Rothamsted Experimental Station, UK). Predicted mean scores were calculated for each line using the GLM for use in the QTL analysis.

## Results

### Genetic map

The genetic map of chromosome 7D in the Hobbit sib (HS) × Hobbit sib/VPM7D (HS/VPM7D) recombinant population (RVPM lines) published by Chapman et al. (2008) has been updated in this work. A new set of 20 7D-specific KASP markers (7D_KASP1 to 7D_KASP20) were developed from the Axiom Wheat HD Genotyping Array (Winfield et al. 2016). These markers are based on 7D-specific SNPs able to distinguish between the HS and HS/VPM7D parents, and they were chosen on the basis of their distribution across the 7D chromosome. Moreover, nine SSRs, namely *Xbarc97*, *Xgdm67*, *Xgdm86*, *Xgdm150*, *Xgwm37*, *Xgwm428*, *Xwmc14*, *Xwmc221* and *Xwmc273* and two biochemical markers, *RC3* and *Amy*-*D2*, were included in the analysis and carried over from Chapman et al. (2008).

**Fig. 1:**
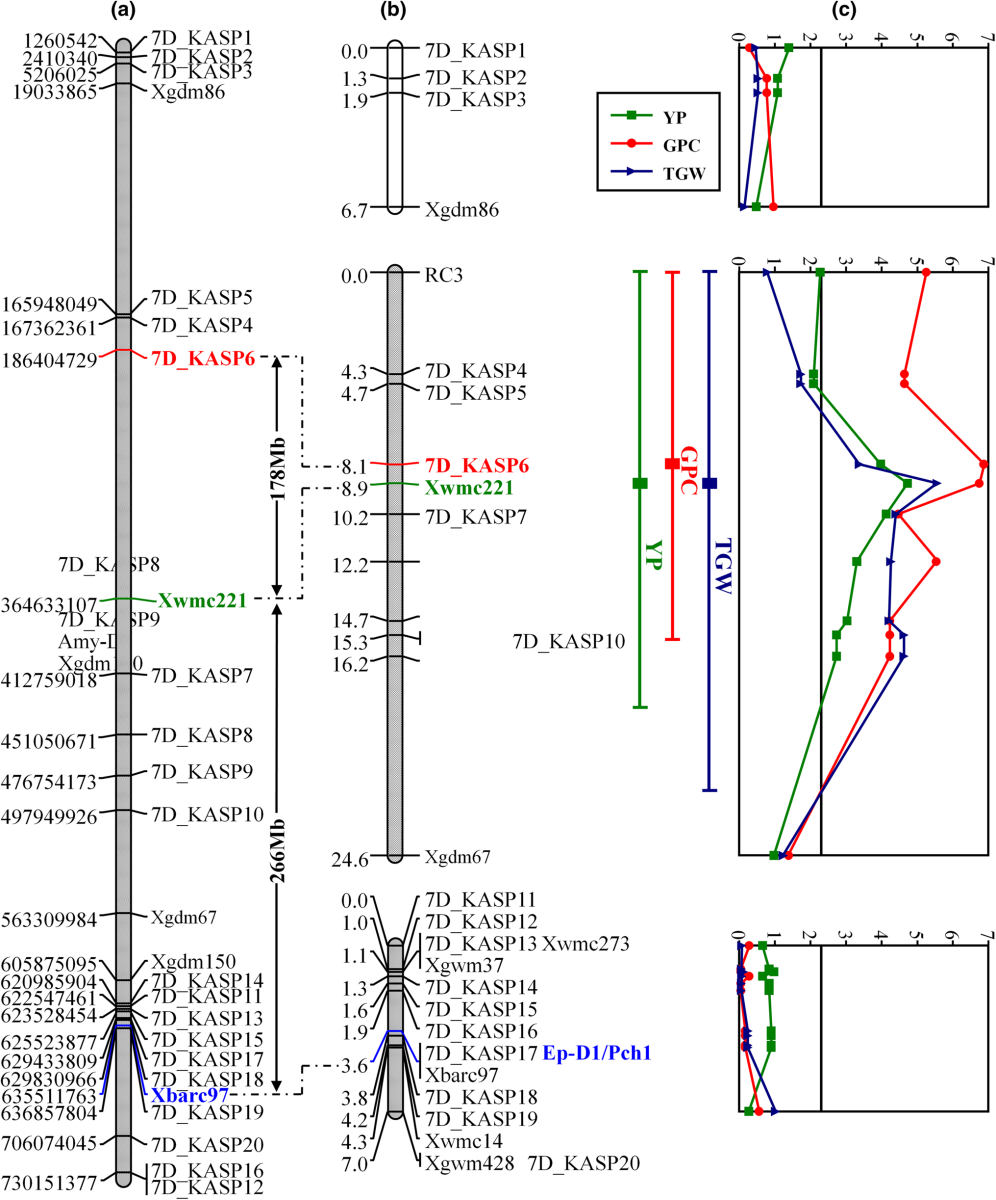
Physical and genetic map of chromosome 7D in the HS × HS/VPMD7D population (**a**, **b**) aligned to the LOD profile of the QTL interval mapping analysis of 4 years trials combined data for yield and GPC, and of two years trials for TGW (**c**). QTL positions are shown to the right of the genetic maps by bars that indicate areas on the map with a LOD score greater than the significance threshold of 2.3. **c** Shows line RVPM003 which possesses the *Ae. ventricosa* alleles for *7D_KASP6* (peak marker for GPC) and *Xbarc97* (*Pch1*) but has the Hobbit sib allele at *Xwmc221* (associated with positive yield)

All these markers are listed in Table 1 along with primer sequences, corresponding BA/BS SNP code and SNP genomic location on the reference genome where identified. These KASPs were used to genotype the 90 RVPM lines of the HS × VPM7D population, and a new genetic map of the 7D chromosome, spanning a total of 38.8 cM and divided into three linkage groups (LG1, LG2, LG3), was produced (Fig. 1b).

**Table 1:** Summary of markers used for mapping YP and GPC in the HS × HS/VPM7D population

Marker name	BA/BS SNP code	Marker type	Primer	Genomic location
7D_KASP1	BA00229805	KASP	cgccaaaccgatcattccC	7D: 1260542
			cgccaaaccgatcattccT	
			cgctaggttacttccctgtg	
7D_KASP2	BA00123654	KASP	ggccgctgttattgctacaG	7D: 2410340
			ggccgctgttattgctacaT	
			gtgtttgcagaatctctatcgg	
7D_KASP3	BA00820637	KASP	gagtgcagggttcagctC	7D: 5206025
			gagtgcagggttcagctG	
			atgatccgccgccccaac	
Xgdm86	–	SSR	ggtcaccctctcccatcc	7D: 19033865–19034000
			ggcgctccattcaatctg	
7D_KASP4	BA00159833	KASP	cagaagagtcagtgacagaagcaG	7D: 167362361
			cagaagagtcagtgacagaagcaT	
			agatataagaacgacaccaaactga	
7D_KASP5	BA00870707	KASP	gctaactacagagagcaccacaG	7D: 165948049
			gctaactacagagagcaccacaA	
			cttagcctgcgattacattgctgc	
7D_KASP6	BA00509217	KASP	tgcacgaaatcgaccatgtA	7D: 186404729
			tgcacgaaatcgaccatgtC	
			cgaaggcgctctcggtaat	
Xwmc221	–	SSR	acgataatgcagcggggaat	7D: 364633107–364632935
			gctgggatcaagggatcaat	
7D_KASP7	BA00181566	KASP	tgaaccgtggatctattgtgcG	7D: 412759018
			tgaaccgtggatctattgtgcA	
			ccgttaaatcagcagcttaatcc	
7D_KASP8	BS00180865	KASP	agaaggcaaatatgttgtagatcttgA	7D: 451050671
			agaaggcaaatatgttgtagatcttgC	
			caactcttgttgaaggggttatctttgta	
7D_KASP9	BA00236459	KASP	ctggggcagcgacatggA	7D: 476754173
			ctggggcagcgacatggG	
			ctgacgctcggcttcgga	
7D_KASP10	BA00386263	KASP	cagtgtgttcgccttagatgtaC	7D: 497949926
			cagtgtgttcgccttagatgtaT	
			accggaattagacaaactgagac	
Xgdm67	–	SSR	aagcaaggcacgtaaagagc	7D: 563309984–563310113
			ctcgaagcgaacacaaaaca	
Xgdm150	–	SSR	actagcctggcagttgatgc	7D: 605875095–605875208
			ccgaccggttcacttcc	
7D_KASP11	BA00121106	KASP	tgtactgccaaaatacgcctG	7D: 622547461
			tgtactgccaaaatacgcctC	
			cggcgaacctcatccact	
7D_KASP12	BA00386209	KASP	gctcacaacacccaccaA	7D: 730153518
			gctcacaacacccaccaT	
			catgaactgaatctgttctgtgg	
7D_KASP13	BS00011507	KASP	tgccttttggtcgaagagttcT	7D: 623528454
			tgccttttggtcgaagagttcG	
			cagccttattcttcttgcttcaagatcaa	
Xwmc273	–	SSR	agttatgtaattctctcgagcctg	–
			ggtaaccactagagtatgtcct	
Xgwm37	–	SSR	acttcattgttgatcttgcatg	–
			cgacgaattcccagttaaac	
7D_KASP14	BA00084318	KASP	tttcgctgcagaacccaaG	7D: 620985904
			tttcgctgcagaacccaaC	
			tcaacaaggaggttcagaatgtt	
7D_KASP15	BA00110518	KASP	cctcgagattgtgctttagattcG	7D: 625523877
			cctcgagattgtgctttagattcA	
			cagttcccaaacaggacca	
7D_KASP16	BA00894181	KASP	atgaccgaggagcatgcT	7D: 730151377
			atgaccgaggagcatgcC	
			aggttcttcatcagcacacG	
7D_KASP17	BA00558682	KASP	gtgttgctattagcattcctccT	7D: 629433809
			gtgttgctattagcattcctccC	
			taacatacacatcaatgctgcttgaggtt	
Xbarc97	–	SSR	gcgccaactacggagctcggagaa	7D: 631323507–631323748
			gcaggatcaaacgtagccatggtg	
7D_KASP18	BA00747082	KASP	agtcaaacctcgcaaacgC	7D: 629830966
			agtcaaacctcgcaaacgT	
			catgagcagcaatgccgac	
7D_KASP19	BA00578168	KASP	atcctcgccttcatgccA	7D: 636857804
			atcctcgccttcatgccG	
			tgGagagcaagatatgtatgttcG	
Xwmc14	–	SSR	acccgtcaccggtttatggatg	7D: 635511763–635512503
			tccacttcaagatggagggcag	
Xgwm428	–	SSR	cgaggcagcgaggattt	
			ttctccactagccccgc	
7D_KASP20	BA00244883	KASP	accgcacatcaaactgagC	7D: 706074045
			accgcacatcaaactgagT	
			ctgcgagtgtggtggggt

### Yield, GPC and TGW: data from individual trials

Examining each field trial individually, yield QTLs were identified in four of the 8 trials. In three of these locations, the QTL was identified in the central linkage group (LG2) (LOD ranging from 3.1 to 10.6 and explained variance ranging from 26.3 to 57.3%), whereas in 1 year (2016_RAGT_SD), the QTL peak was in the distal linkage group (LG3) (Table 2 and Online Resource 1, 2b, 2f and 2h).

**Table 2:** Summary of QTL interval mapping of every single year field trial data for yield, GPC and TGW. Significance threshold is 2.3

Trial	Protein content			Yield			Thousand grain weight		
	Locus name	%Expl. var.	− log10 (*P*)	Locus name	%Expl. var.	− log10 (*P*)	Locus name	%Expl. var.	− log10 (*P*)
2014_JIC	RC3	40.7	5.8	None	–		Amy-D2	32.0	3.9
2015_Limagrain	7D_KASP4	26.9	3.3	Xwmc221	57.3	10.6	n.d.	n.d.	n.d.
2015_RAGT_WH	none	–	–	None	–	–	n.d.	n.d.	n.d.
2015_RAGT_SF	7D_KASP6	26.6	3.0	None	–	–	n.d.	n.d.	n.d.
2016_Limagrain	Xgdm86	26.7	4.1	None	–	–	n.d.	n.d.	n.d.
2016_RAGT_BT	Xwmc221	67.3	11.7	Amy-D2	26.3	3.1	n.d.	n.d.	n.d.
2016_RAGT_SD	Xwmc221	55.7	8.7	7D_KASP11	24.2	2.9	n.d.	n.d.	n.d.
2017_JIC	7D_KASP4	38.4	5.1	7D_KASP6	47.0	6.8	Xwmc221	45.5	6.6

For GPC, a QTL was observed in 7 of the 8 field trials. In six of these locations, the QTL peak was within the central linkage group (LG2) (LOD scores ranging from 3.0 to 11.7; explained variance ranging from 26.9 to 67.3%), whereas in 1 year (2016_Limagrain) the QTL peak was in the proximal linkage group (LG1) (Table 2 and Online Resource 1, 2a-2d and 2f-2h).

Finally, a TGW QTL was identified in both trials where this trait was assessed (2014_JIC and 2017_JIC). In both cases, the QTL peak was located in the middle linkage group (LG2) (LOD was 3.9 and 6.6; explained variance was 32.0 and 45.5, respectively) (Table 2 and Online Resource 1, 2a and 2h).

### Yield, GPC and TGW: data combined from all trials

To obtain an overall picture of the effect of the 7D^V^ introgressed segment on yield and protein content, predicted means of yield, GPC and TGW from each field trial were combined and a new interval mapping QTL analysis was carried out. This overall analysis revealed that *7D_KASP6* (LOD = 6.88) is the marker most significantly associated with GPC explaining 46.6% of the observed phenotypic variance with the beneficial allele being contributed by *Ae. Ventricosa*. QTL interval laid between markers *RC3* and *Xgdm150* and spanning a region of 16.2 cM (Table 3 and Fig. 1c). The most significantly associated marker for yield was *Xwmc221* with a LOD of 4.73 and explaining 40.1% of the observed phenotypic variance. The high value allele for this QTL was derived from HS and was located between markers *7D_KASP6* and *Xgdm150*, spanning 8.1 cM (Table 3 and Fig. 1c). For TGW, the combined 2-year data identified a QTL with *Xwmc221* as the most significantly associated marker (LOD = 5.56), explaining 35.9% of the observed variance and with the QTL region spanning 8.1 cM between markers *RC3* and 7D_*KASP10*. As for the yield QTL, the high value allele was derived from HS.

**Table 3:** Summary of the QTL interval mapping analysis of 4 years combined data for yield and GPC, and 2 years combined data for TGW on chromosome 7D. Significance threshold is 2.3

Locus	Genetic position	Protein	Yield	TGW
		LOD* score	LOD* score	LOD* score
RC3	0.00	5.25	2.27	0.79
7D_KASP4	4.28	4.64	2.09	1.73
7D_KASP5	4.72	4.64	2.09	1.73
7D_KASP6	8.13	6.88	3.97	3.37
Xwmc221	8.94	6.75	4.73	5.56
7D_KASP7	10.24	4.47	4.13	4.40
7D_KASP8	12.17	5.16	3.30	4.26
7D_KASP9	14.66	4.23	3.03	4.21
Amy-D2	15.32	4.23	2.73	4.63
7D_KASP10	15.32	4.23	2.73	4.63
Xgdm150	16.22	4.23	2.73	4.63
Xgdm67	24.56	1.38	0.97	1.23
	QTL effect	7D_KASP6	Xwmc221	Xwmc221
	%Expl. var.	46.6	40.1	35.9
	High value allele	VPM7D	Hobbit Sib	Hobbit Sib

*LOD score = − Log10(P)

The physical position on the chromosome 7D of all the markers used for the genetic map was identified by comparison with the reference sequence of Chinese Spring wheat (IWGSC 2018) at *Ensembl*Plants (http://plants.ensembl.org/index.html) (Kersey et al. 2018) (Fig. 1 and Table 1). Although this procedure might be biased by the fact that unknown genomic rearrangements may have occurred since *Ae. ventricosa* and wheat diversification, it provides an indication of the physical distances between markers. Consequently, even if the two peak markers for yield and GPC are genetically very close (0.8 cM), they seem to be physically distant being 178 Mb apart. More importantly, the yield and *Pch1* loci seem to be located 266 Mb apart [physical distance calculated from *Xwmc221*, yield peak marker to *Xbarc97*, SSR marker co-segregating with *Pch1* (Chapman et al. 2008)]. This provides good evidence that the negative yield effect from *Ae. ventricosa* is not physically linked to *Pch1* (Table 1).

A further confirmation of the reasonable physical distances among Yield, GPC and *Pch1* peak markers was the identification of a recombinant RVPM line (line RVPM 3) in which a double recombination had occurred, and the line contained *Pch1* and the *7D_KASP6*, (GPC QTL peak marker) allele from *Ae. ventricosa*, but did not possess the *Xwmc221* (Yield QTL peak marker) allele from Hobbit sib. This line has been backcrossed to Hobbit Sib for fine mapping of the yield and GPC QTL to establish whether the association between the two traits can be separated (data not shown).

A regression analysis was performed classifying individuals of the RVPM population in two groups to quantify the effect of the 7D^V^ introgressed segment on yield and GPC: group A containing all genotypes with the wheat allele for the yield penalty peak marker *Xwmc221* and group B with all genotypes having the *Ae. ventricosa* allele for the same marker. The same process was performed using the GPC peak marker *7D_KASP6* and the GPC peak marker *Xwmc221*. Results showed that the presence of the 7D^V^ segment causes an increase of 2.6% in GPC (*P* = 0.01) and a 3.5% reduction in yield (*P* = 0.01), if the combined data of the 4 years trial were considered. A reduction of 5.8% of the TGW was also observed when combined data of 2 years (2014 and 2017) were considered. Significant differences in protein content and yield were also identified in each individual field trial (Online Resource 3).

## Discussion

In the present study, KASP markers were integrated into the SSR-based map of the 7D chromosome from RVPM lines published by Chapman et al. (2008). The new version of the genetic map, although still divided into three linkage groups, proved effective for the purpose of this study. The relationship between the negative yield effect and *Pch1* has long been uncertain, but early reports suggested that the addition of the entire 7D^V^ chromosome could reduce yields by approximately 8% (Worland et al. 1990). Some studies have concluded that the presence of *Pch1* is responsible for any negative impact on yield (Kwiatek et al. 2016), while other studies noted that an effect only occurred in some instances (Koen et al. 2002). In an early study using the same population as used in the current work, but lacking a detailed genetic map, a negative impact on yield was associated with the *Pch1* locus on both drilled and spaced planting plot trials while a second, more pronounced effect was identified nearer the centromere only in the drilled plot trial (Worland et al. 1990). The centromeric region was also associated with an increased tiller number in lines carrying the *Ae ventricosa* alleles (Worland et al. 1990). In the current study, no evidence was found for any association between the *Pch1* locus and yield.

A negative influence of the *Ae. ventricosa* introgression on yield was observed in some trials of this work, but this was associated with a region close to the centromere rather than *Pch1,* which is located close to the telomere. When an interval mapping QTL analysis was performed combining results from the 4 years of trials, a moderate effect QTL was identified which was responsible for a yield reduction of 3.1%. Worland and colleagues (1990) associated this region with increased tiller number and speculated that the yield reduction was due to reduced grain size as a consequence of increased grain number. In the current study, although the yield QTL interval was coincident with that for grain size (TGW), an effect on yield was only observed in the 2017 trial but not in 2014. We speculate that the yield penalty effect may be associated with the smaller grain size, but that other factors such as tiller number may be important in determining whether a negative impact on yield occurs.

The fact that the yield QTL was identified only in four out of the eight field trials (in three of the four years) suggests that the yield QTL is not stable, and its effect is possibly affected by environmental factors. More importantly, our results clearly demonstrate that the yield QTL and *Pch1* are located on different linkage groups in the RVPM population, indicating that, in those years where a yield penalty is present, it is not linked to *Pch1*. This also implies that the two effects can be recombined as suggested by the large physical distance of 266 Mb between the yield QTL peak marker and *Pch1* (Chapman et al. 2008) in the Chinese Spring reference genome, and the fact that we identified 40 recombinants across this interval within the 90 lines of the population.

Based on these results, *Pch1*-mediated eyespot resistance can be selected for without the negative yield QTL within the wider introgression region. Burt and Nicholson (2011) showed that most of the *Pch1*-containing European wheat cultivars they examined possess a relatively large portion of the *Ae. ventricosa* original segment and they speculated that the negative yield effect must have been compensated by other factors. The current study reveals that as evidenced by the association between the yield penalty and marker *Xwmc221* the deleterious portion of the 7D^V^ introgression affecting yield has been removed by natural recombination in these recent *Pch1*-containing cultivars.

In addition to the 7D^V^ chromosome, the line VPM1 also possesses a second introgression (2N^S^) from *Ae. ventricosa* onto chromosome 2A (Jahier et al. 2001). It is conceivable that some of the negative effect on yield may have been as a result of varieties carrying both the *Pch1* 7D^V^ and 2N^S^ introgressions and that subsequent loss of the 2N^S^ segment has reduced the negative impact.

Until now, the relative positions of *Pch1* and the yield and GPC traits were unknown. In the present study, the region of 7D^V^ responsible for the increased GPC in wheat associated with the *Ae. ventricosa* introgression was identified and shown to confer a relative increase of 2.6% in respect to the wheat GPC (Hobbit sib). This increase is consistent with the data published by Bogard et al. (2010) and Groos et al. (2004). Moreover, combining genotyping and GPC data collected in our field trials, this effect has been mapped for the first time to a centromeric region of chromosome 7D. A QTL for GPC was identified in every year except for the 2015_RAGT_WH trial, indicating that the GPC effect located on the introgressed 7D^V^*Ae. ventricosa* segment is highly stable, unlike the yield penalty effect. The GPC increase QTL is on the linkage group 2 of the HS × HS/VPM7D map 0.8 cM proximal to the yield QTL. Although *Ae. ventricosa* and wheat shared a common ancestor and unknown genomic rearrangements may have occurred since their diversification, this genetic distance corresponds to a large physical distance of 178 Mb on the Chinese Spring reference genome (Table 1 and Fig. 1a). Although the yield and GPC QTL intervals are large and overlap, our data revealed that overall the GPC peak marker located proximal to the yield peak marker. This provides encouragement that it will be possible to separate the yield and GPC regions from one-another and produce varieties carrying both *Pch1* and the GPC QTL.

A new large HS × HS/VPM7D population has been produced, and this will be screened to identify additional recombinant lines in this region to confirm whether the yield and GPC traits can be separated. In addition, our study indicates the potential value of positively selecting the *Ae. ventricosa* allele at the *7D_KASP6* marker for GPC improvement in future wheat breeding programs. Line RVPM003 within the population contains a double recombination and possesses the *Ae. ventricosa* allele for *7D_KASP6* along with *Pch1* but has the Hobbit sib. allele at *Xwmc221* associated with positive yield. This line may have both the positive 7D^V^ associated traits (*Pch1* and high GPC) while lacking the negative yield effect, although more work is required to confirm this.

In conclusion, *Pch1* is a potent and, to date, durable resistance gene effective against both *Oculimacula* species that cause eyespot. The introgression into wheat of the large 7D^V^ chromosome segment carrying this gene from a wild relative is also associated with other positive and negative ‘linkage-drag’ effects. The dissection and characterization of the 7D^V^ segment for yield and GPC traits performed in this study have shown that the yield penalty is not a stable effect and can be separated from *Pch1*. A stable increased GPC effect has been associated with the *Ae. ventricosa* segment which may be useful in future wheat breeding programs. This trait is also unlinked to *Pch1*, and we have identified markers that can be used to select for *Pch1* and increased GPC and against the yield penalty effect.

## Electronic supplementary material

Below is the link to the electronic supplementary material.

Supplementary material 1 (XLSX 20 kb)

Supplementary material 2 (PDF 222 kb)
