# Supplementary material for: Yield reduction historically associated with the Aegilops ventricosa 7DV introgression is genetically and physically distinct from the eyespot resistance gene Pch1
Source: Theor Appl Genet. 2019 Dec 13;133(3):707–17. doi: 10.1007/s00122-019-03502-1 (PMC7021663; doi:10.1007/s00122-019-03502-1)
Supplement: Supplementary file 2 — Supplementary material 2 (PDF 222 kb) [file 122_2019_3502_MOESM2_ESM.pdf]

Supplementary material

Yield reduction historically associated to the *Aegilops ventricosa* 7DV introgression is genetically and physically distinct from the eyespot resistance gene *Pch1*

Theoretical and Applied Genetics

M. Pasquariello1, S. Berry2, C. Burt3, C. Uauy1, P. Nicholson1

1John Innes Centre, Norwich Research Park, Norwich, UK
2Limagrain UK, Limagrain UK Ltd, Rothwell, Market Rasen, Lincolnshire LN7 6DT, UK
3RAGT Seeds, Grange Road, Ickleton, Essex CB10 1TA, UK

Corresponding author
P. Nicholson,
John Innes Centre, Norwich Research Park,
Colney, Norwich NR4 7UH, UK
e-mail: paul.nicholson@jic.ac.uk
Phone: +44 (0)1603 450616
Fax: +44 (0)1603 450045

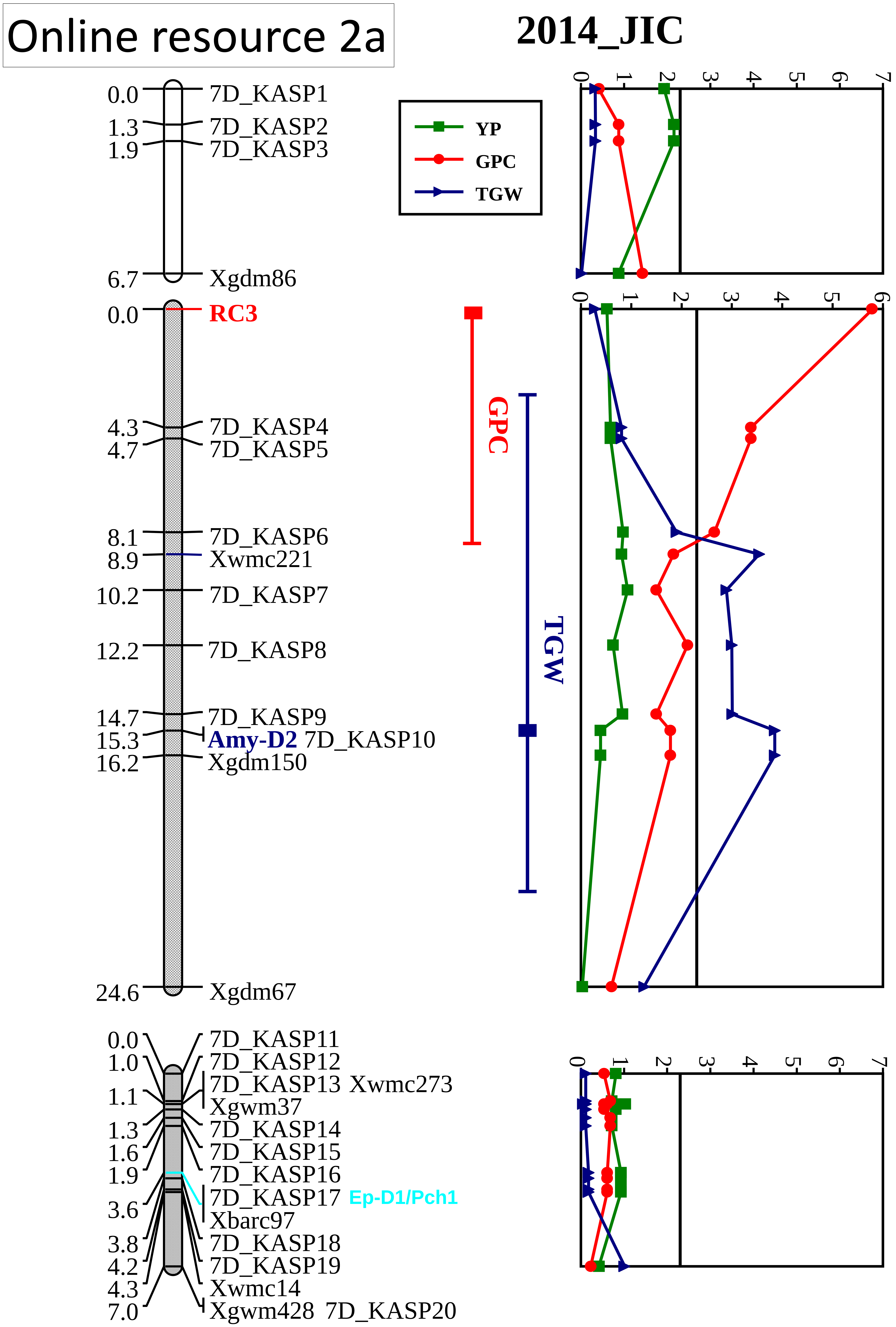

Online Resource 2a: Genetic map of chromosome 7D in the HS x HS/VPMD7D population aligned to the LOD profile of the QTL interval mapping analysis of 2014_JIC field trial for YP, GPC and TGW. No QTL has been identified for YP.

Online resource 2b

2015_Limagrain

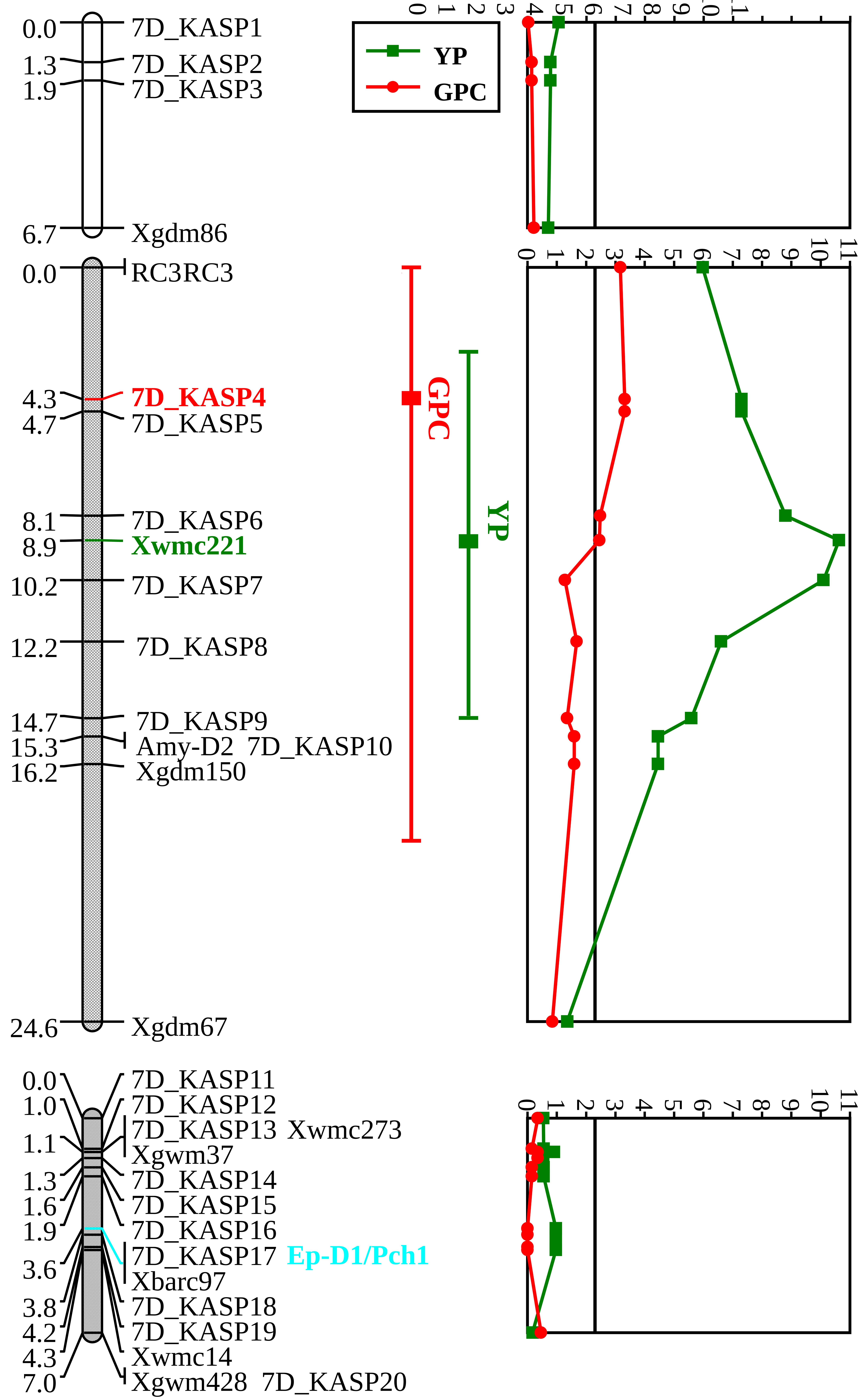

Online Resource 2b: Genetic map of chromosome 7D in the HS x HS/VPMD7D population aligned to the LOD profile of the QTL interval mapping analysis of 2015_Limagrain field trial GPC and YP. Na data available for TGW.

Online resource 2c

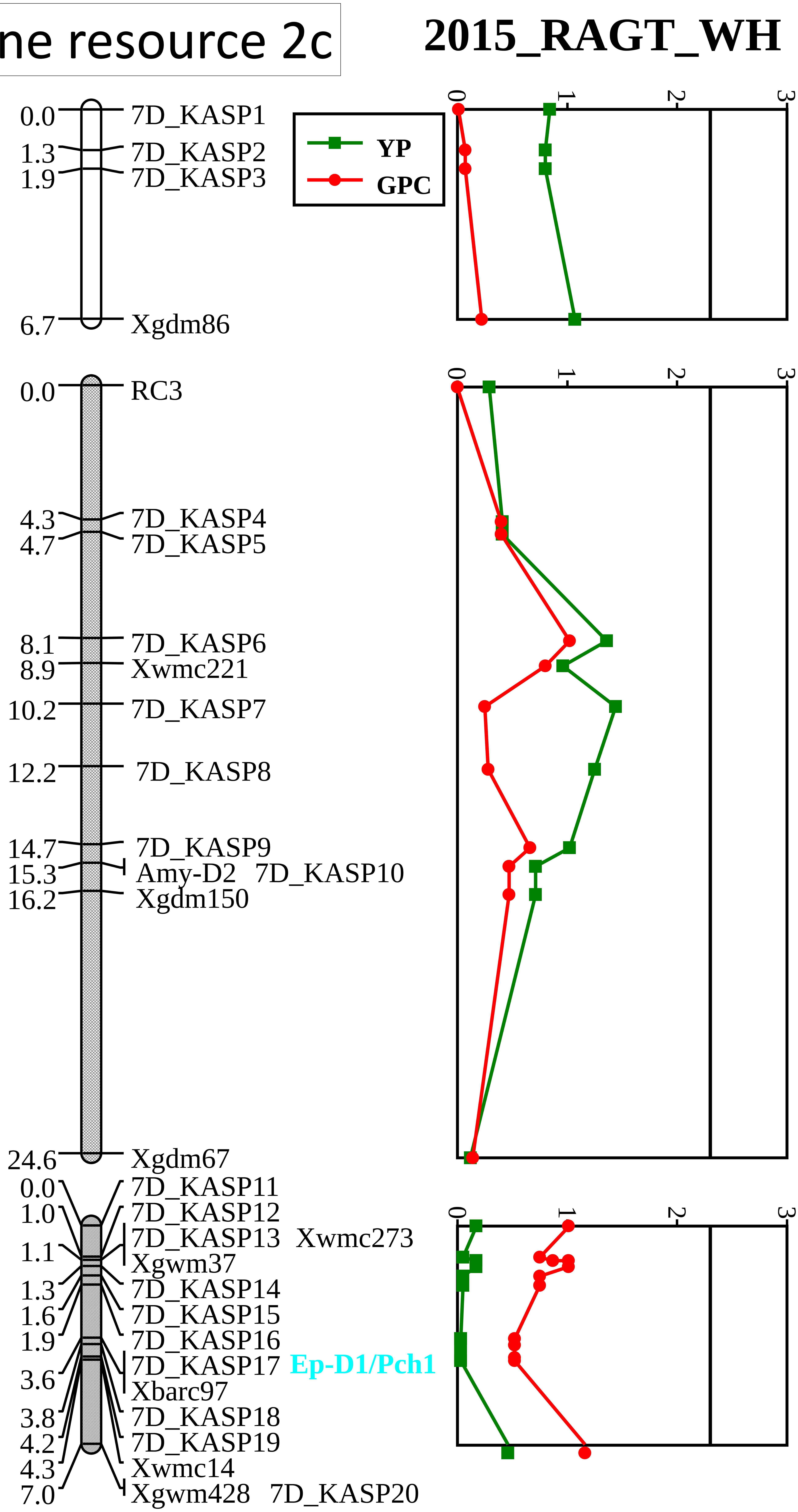

Online Resource 2c: Genetic map of chromosome 7D in the HS x HS/VPMD7D population aligned to the LOD profile of the QTL interval mapping analysis of 2015_RAGT_WH field trial GPC and YP. No QTL effect was identified.

Online resource 2d

2015_RAGT_SF

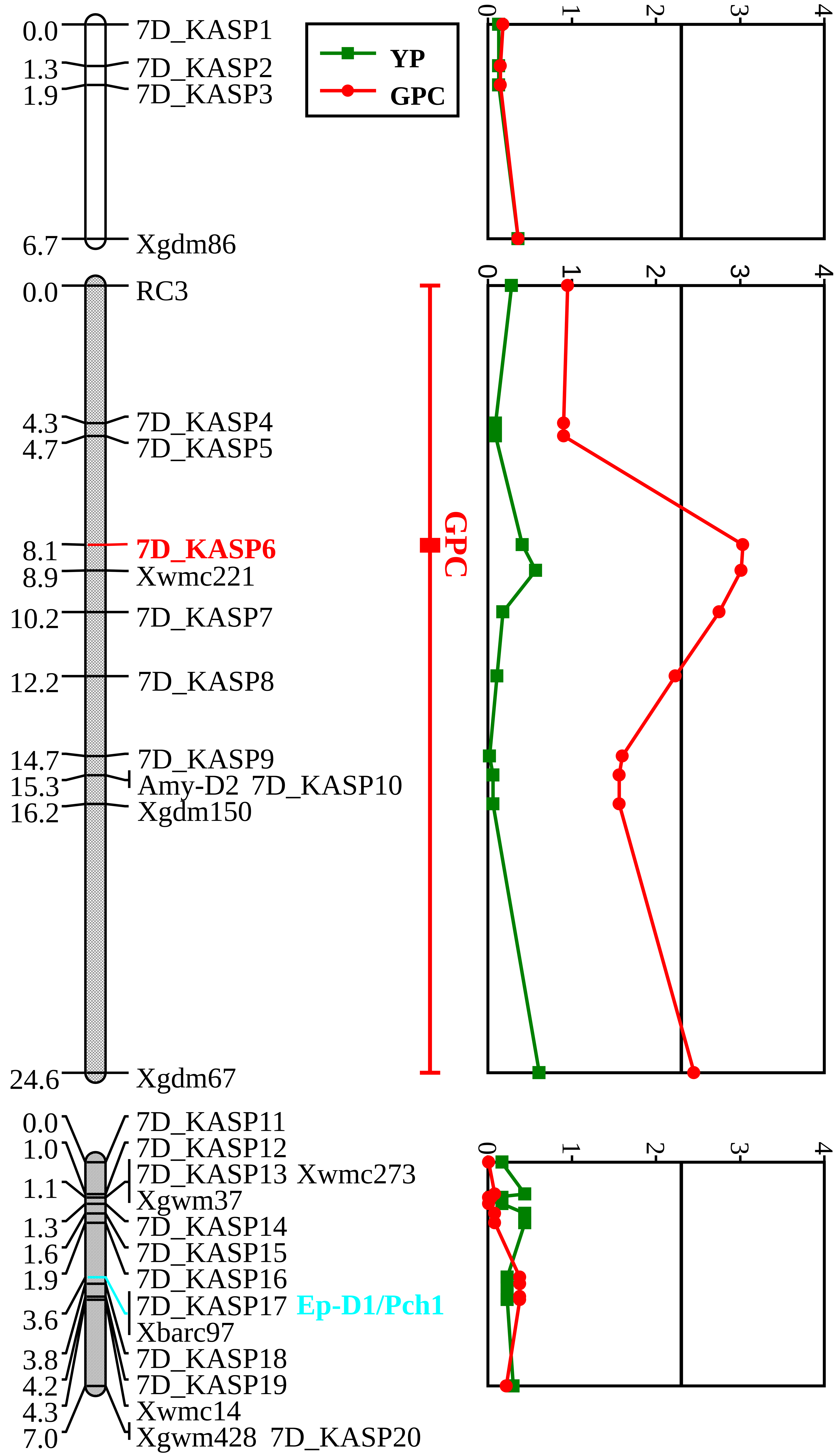

Online Resource 2d: Genetic map of chromosome 7D in the HS x HS/VPMD7D population aligned to the LOD profile of the QTL interval mapping analysis of 2015_RAGT_SF field trial GPC and YP. Na data available for TGW.

Online resource 2e

2016_Limagrain

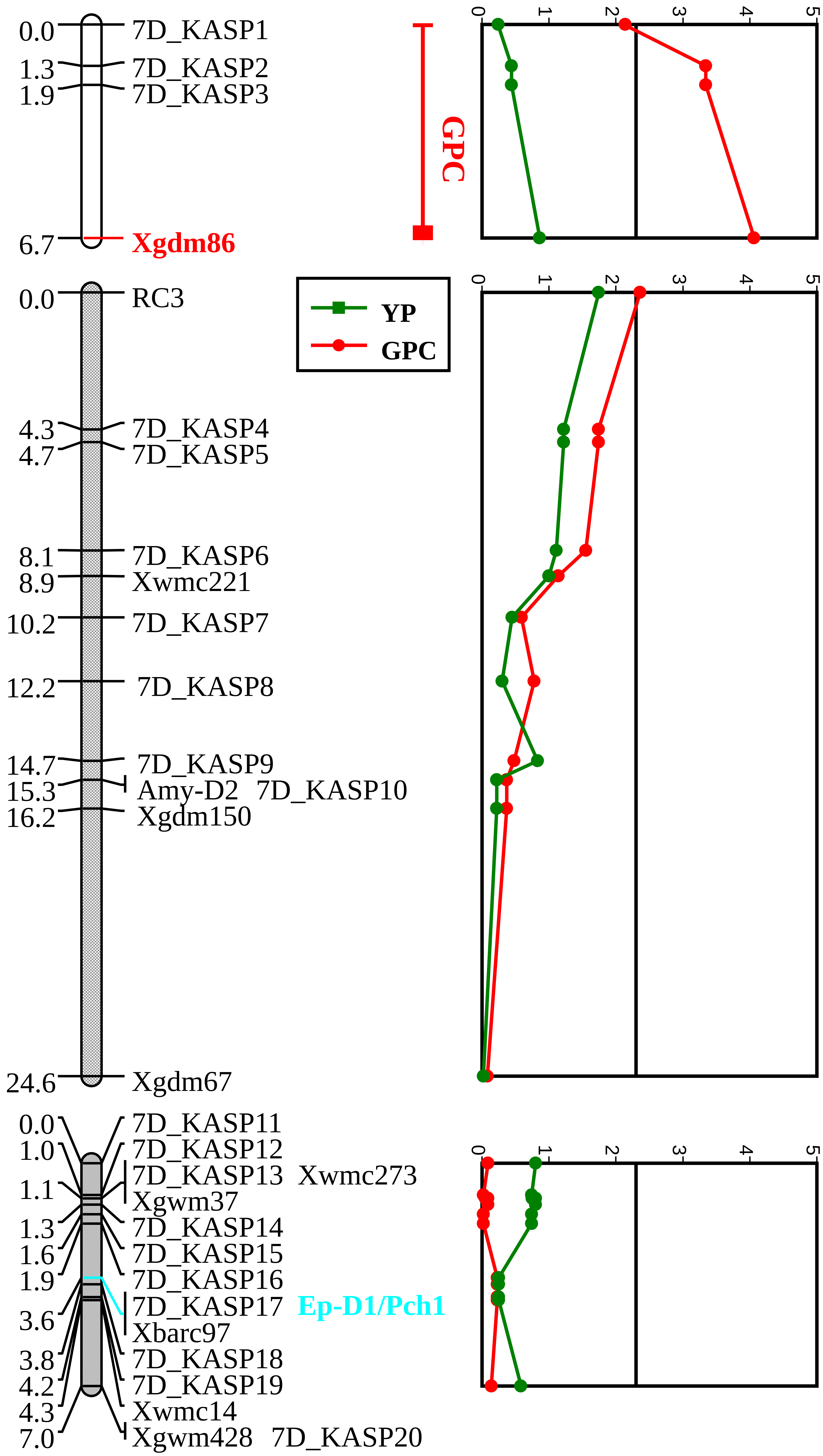

Online Resource 2e: Genetic map of chromosome 7D in the HS x HS/VPMD7D population aligned to the LOD profile of the QTL interval mapping analysis of 2016_Limagrain field trial GPC and YP. Na data available for TGW.

Online resource 2f

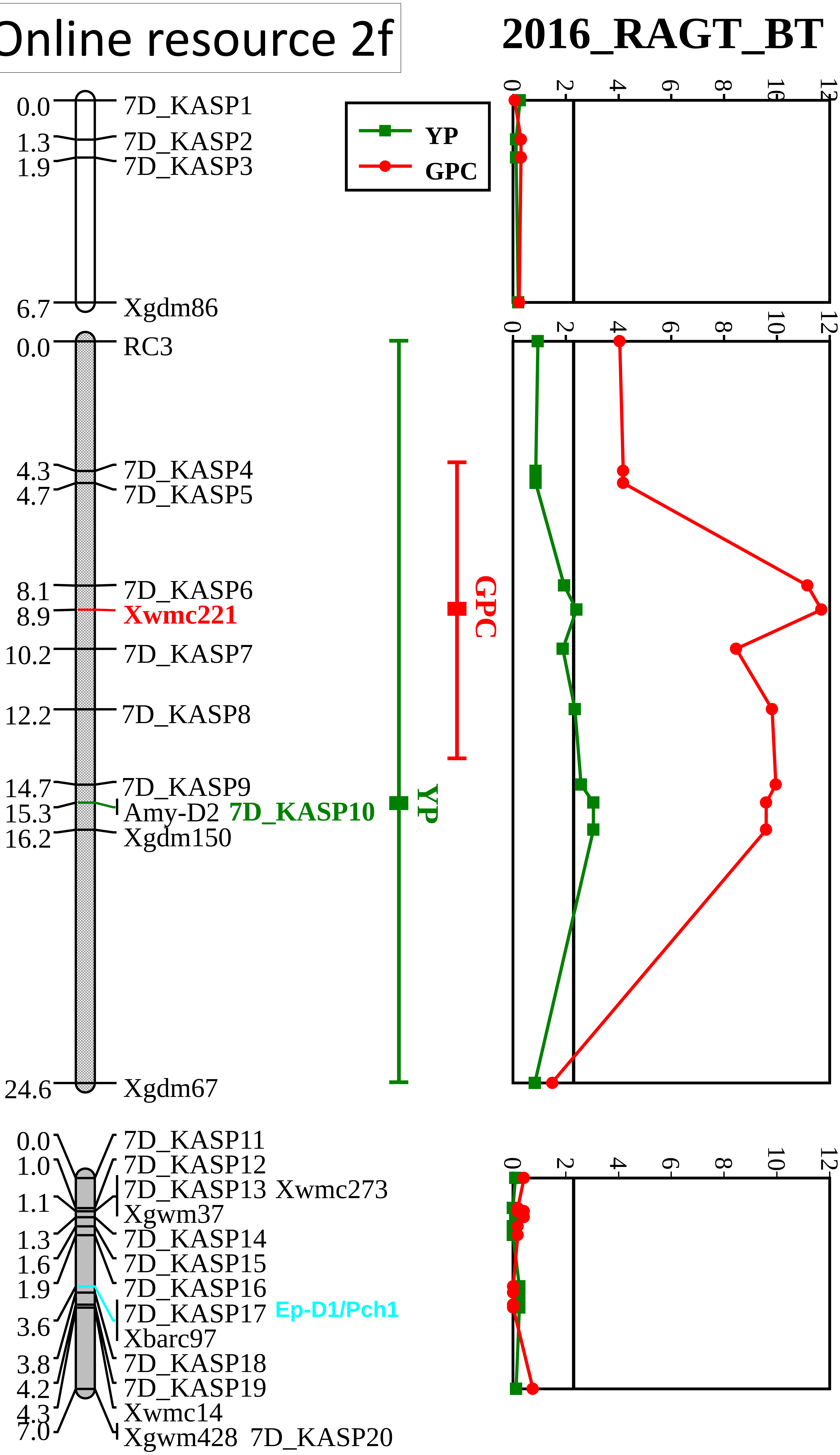

Online Resource 2f: Genetic map of chromosome 7D in the HS x HS/VPMD7D population aligned to the LOD profile of the QTL interval mapping analysis of 2016_RAGT_BT field trial GPC and YP. Na data available for TGW.

Online resource 2g

2016_RAGT_SD

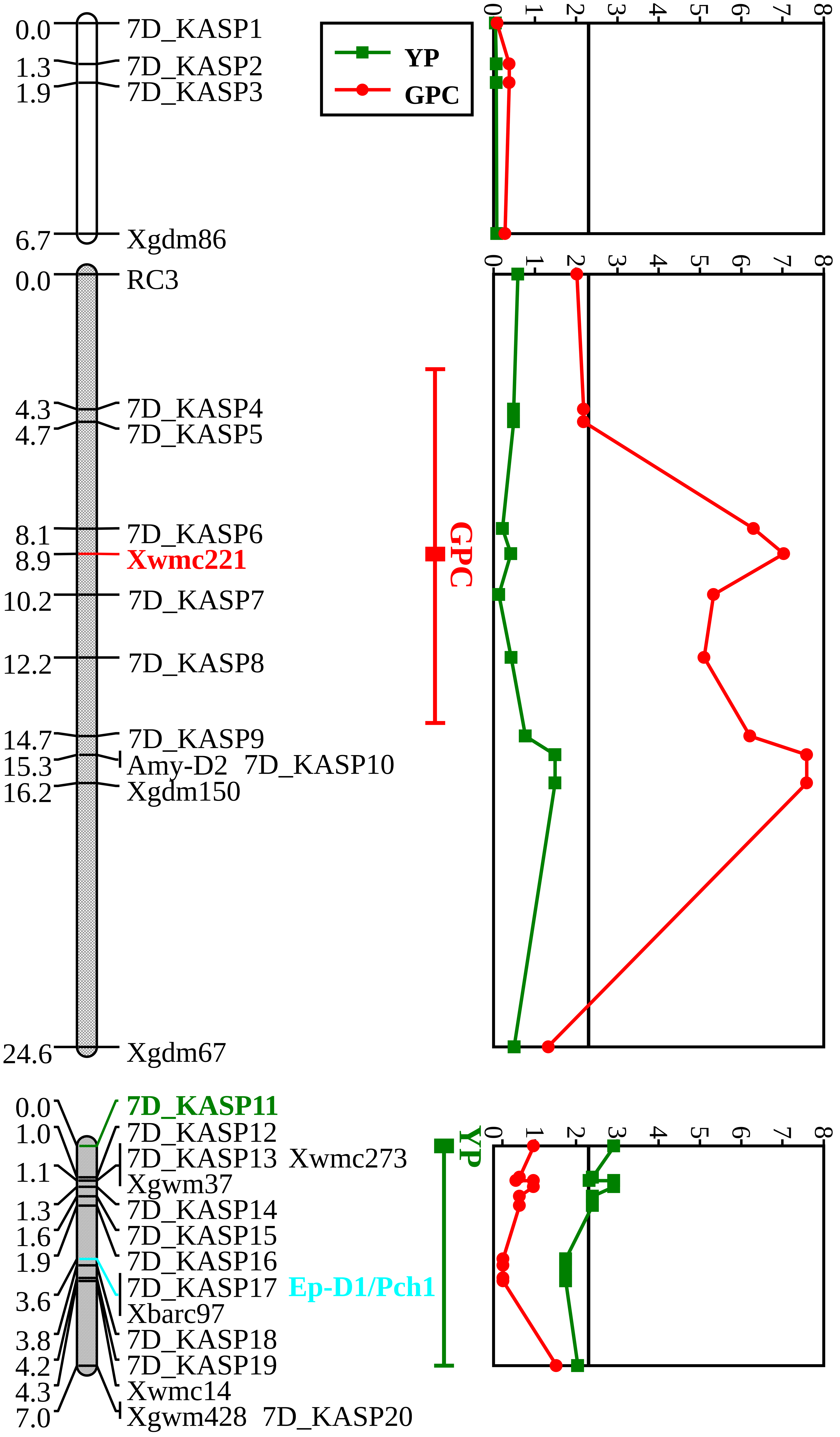

Online resource 2h

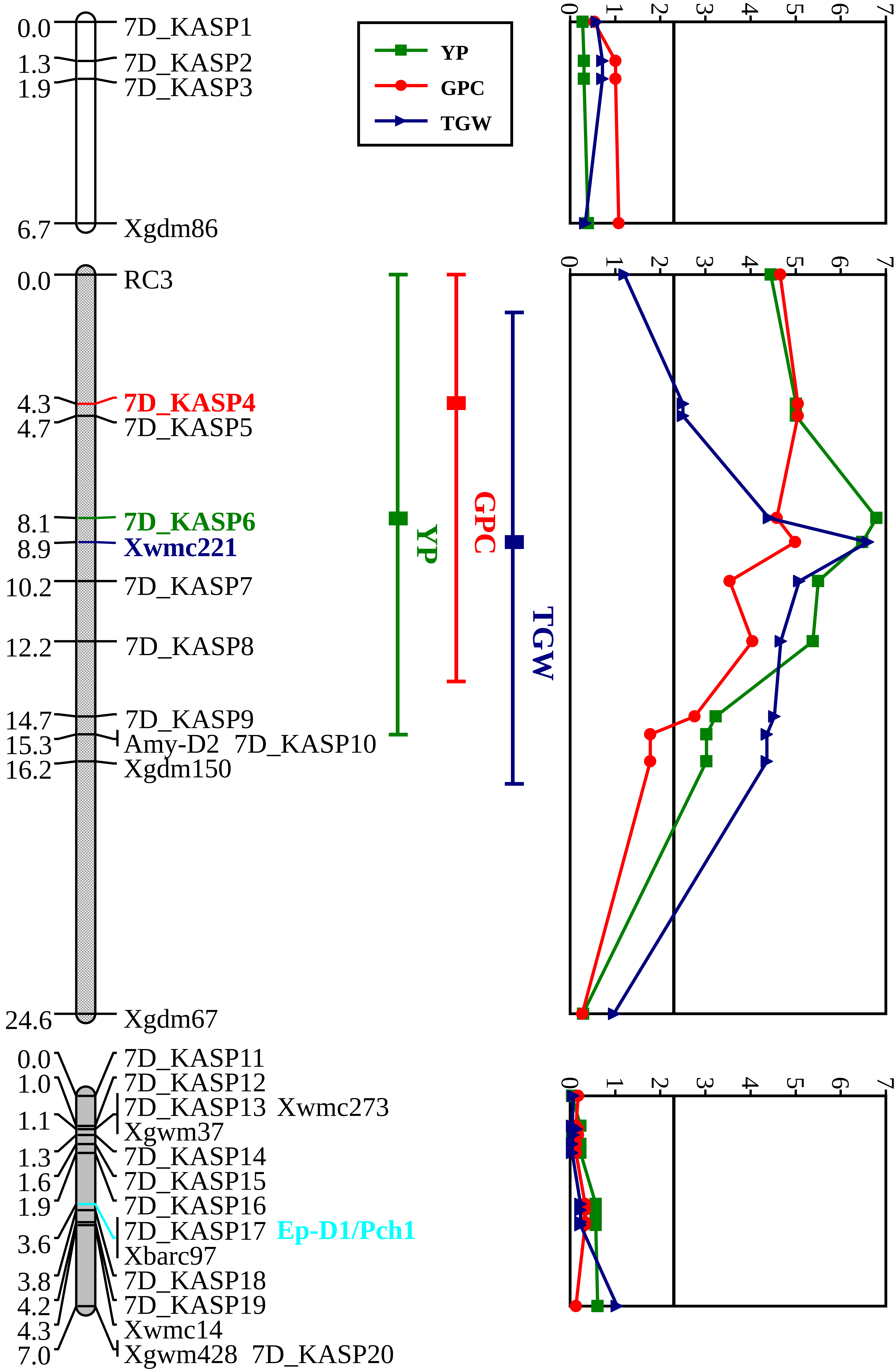

Online Resource 2h: Genetic map of chromosome 7D in the HS x HS/VPMD7D population aligned to the LOD profile of the QTL interval mapping analysis of 2017_JIC field trial for YP, GPC and TGW.
